# Predictive factors for the development of macular neovascularization in chronic central serous chorioretinopathy

**DOI:** 10.1038/s41598-025-17499-9

**Published:** 2025-09-01

**Authors:** Marie Kitano, Shuichiro Aoki, Kohdai Kitamoto, Ryo Terao, Tatsuya Inoue, Ryo Obata, Keiko Azuma

**Affiliations:** 1 Department of Ophthalmology, Graduate School of Medicine, The University of Tokyo, 7-3-1 Hongo, Bunkyo-ku, Tokyo, 113-8655 Japan; 2 Department of Ophthalmology, JR Tokyo General Hospital, 2-1-3 Yoyogi, Shibuya-ku, Tokyo, 151-8528 Japan; 3 Department of Ophthalmology and Micro-Technology, School of Medicine, Yokohama City University, 4-57 Urafune-cho, Minami-ku, Yokohama, 232-0024 Kanagawa Japan; 4 Department of Ophthalmology, Toranomon Hospital, 2-2-2 Toranomon, Minato-ku, Tokyo, 105-8470 Japan

**Keywords:** Predictive factors, Macular neovascularization, Chronic central serous chorioretinopathy, The cumulative duration of the persistence of SRD, Numerically defined DLS, Eye diseases, Macular degeneration, Retinal diseases

## Abstract

This single-center, retrospective study analyzed the development of macular neovascularization (MNV) in patients with chronic central serous chorioretinopathy (CSCR) during a treatment-free follow-up period and the factors associated with its development. In total, 236 patients (280 eyes, 149 males and 87 females, mean age 55.3 ± 13.9 years) with treatment-naïve CSCR with no MNV detected on multimodal imaging who were observed for > 6 months were assessed. The association between age, sex, the presence of MNV, central choroidal thickness (CCT), the presence of the double-layer sign (DLS), and the cumulative duration of the persistence of serous retinal detachment (SRD) was also analyzed to determine the visual outcome before the onset of MNV. Twenty-eight (10%) eyes developed MNV over a mean follow-up period of 48.3 ± 43.3 months. The persistence of SRD for ≥ 6 months (*p* = 0.003) and presence of DLS measuring ≥ 1000 μm in length and < 100 μm in height (*p* = 0.040) exhibited significant associations with the development of MNV. Furthermore, the persistence of SRD for ≥ 6 months showed a significant association with logMAR visual acuity loss of ≥ 0.3 (*p* = 0.0009). Thus, the persistence of SRD for ≥ 6 months and numerically defined DLS were identified as predictors for the development of MNV in patients with CSCR.

## Introduction

Central serous chorioretinopathy (CSCR), characterized by the presence of ≥ 1 serous retinal detachments (SRDs), may be accompanied by serous pigment epithelial detachment (PED). Focal leaks from gaps in the retinal pigment epithelium (RPE), often present in association with choroidal hyperpermeability, lead to CSCR. Thus, CSCR is a subretinal serous retinal reservoir created by the disruption of the outer blood-retinal barrier and pumping of RPE. This process occurs secondary to the accumulation of exudate in the inner choroid owing to increased permeability and blood stasis in the choroidal vasculature^1,2^. CSCR typically resolves spontaneously within 3–4 months; the persistence of SRD beyond 4–6 months is defined as chronic CSCR.

Chronic CSCR is multifocal, bilateral, and recurrent in most cases. Notably, RPE atrophy and retinal thinning result in poor visual prognosis in patients with chronic CSCR^3,4^. The treatment choices for chronic CSCR with persistent exudative changes include laser therapy, photodynamic therapy (PDT), and anti-vascular endothelial growth factor (VEGF) therapy; no treatment is indicated following the resolution of the exudative changes. Recurrence of fluid accumulation following resolution is common. However, a consensus regarding an optimal treatment strategy for patients with repeated recurrence and resolution remains to be established^5^.

Macular neovascularization (MNV), a complication of CSCR that is often associated with pachychoroid neovasculopathy (PNV), causes further deterioration of visual function^6,7^. Previous studies examining the long-term follow-up of CSCR have suggested that the visual prognosis of patients with CSC who develop MNV is poorer than that of those who do not^8^.

Shiragami et al.^7^ reported a significant association between the chronicity of CSCR and the development of MNV^9^. Recent studies have considered CSC a pachychoroid spectrum disease, with some studies evaluating the risk of developing MNV^10,11^. However, the specific mechanisms underlying the development of MNV and the risk factors are poorly understood. An increased incidence of MNV following laser therapy or PDT for CSCs has been reported. This may be attributed to the rupture of the Bruch’s membrane (BM) following laser therapy, choroidal ischemia, and the excessive production of VEGF following PDT^12,13^. Information regarding the risk factors for the development of MNV in patients with untreated CSCR is insufficient. Recent studies have revealed that irregular retinal pigment epithelial elevation on optical coherence tomography (OCT) is an indicator of MNV and exudative risk^14,15^.

The double-layer sign (DLS), defined as the presence of two highly reflective layers underneath^14,16,17^, was initially considered an indicator of polypoidal choroidal vasculopathy (PCV). However, DLS has also been observed in various other conditions, including CSCR. For instance, DLS has been observed in eyes with nonexudative MNV with or without drusen, indicating a risk of future exudation^15^. Conventional dye-based angiography, such as fluorescein angiography (FA) and indocyanine green angiography (ICGA), as well as optical coherence tomography angiography (OCTA), have revealed that MNV is not detected in all cases with DLS. Thus, the pathological significance of DLS without MNV and its predictive value for the risk of developing MNV remains to be elucidated.

Prolonged SRD may result in RPE dysfunction and induce the development of MNV^18^. Notably, a longer cumulative period is associated with a poorer visual prognosis, even in cases with intermittent recurrent exudative changes^8^. However, the correlation between the presence of exudative changes, including intermittent recurrences, and the risk of developing MNV, especially in patients with CSCR who cannot receive treatment and those observed without treatment, is unclear.

The risk factors for the development of MNV, which may have a significant impact on visual prognosis, in patients with CSCR remain to be elucidated, particularly in untreated patients under observation. The development of MNV in patients with CSCR under observation without treatment may be associated with predisposing anatomical factors. The present study aimed to identify these factors.

## Results

**Fig. 1:**
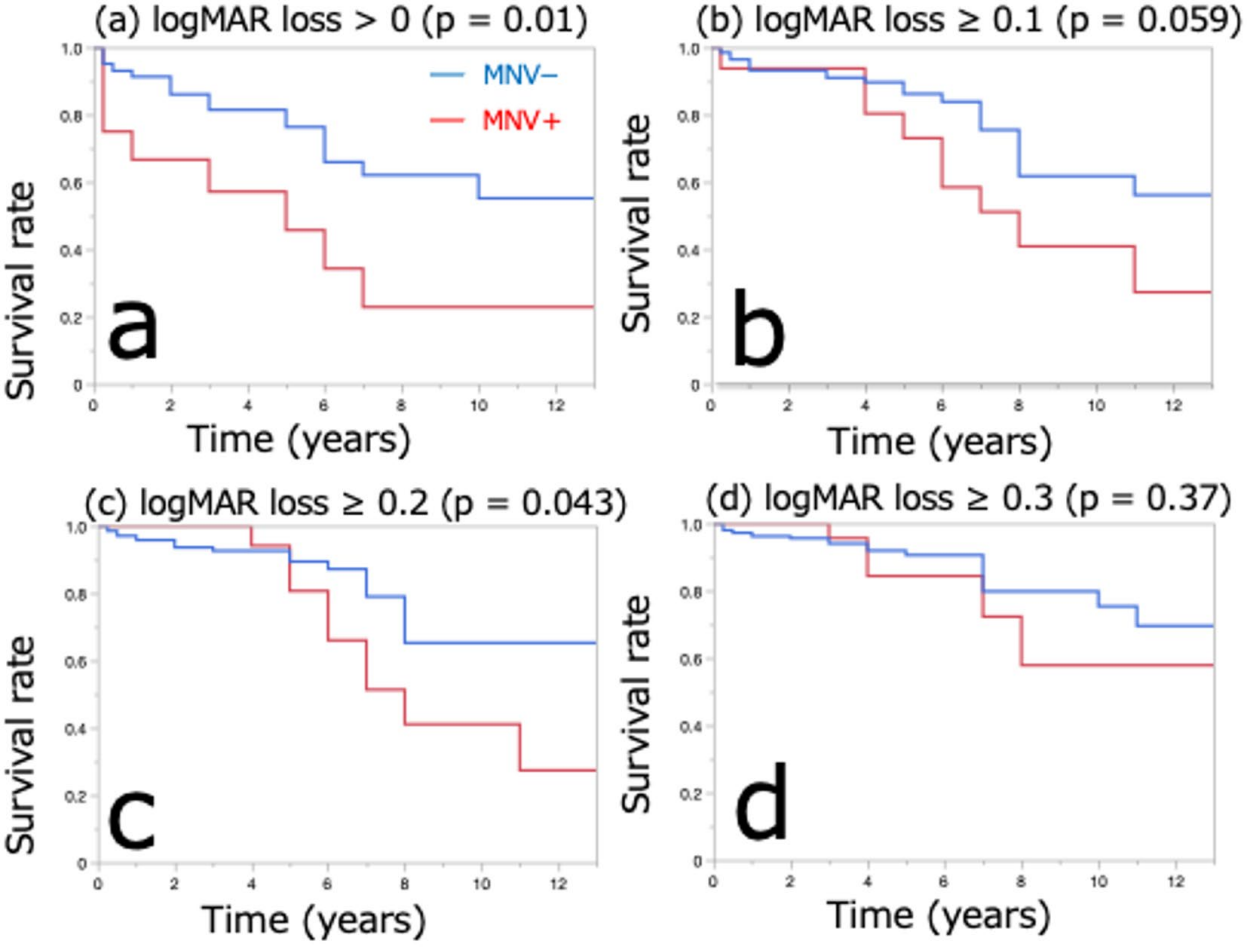
The Kaplan–Meier method was used to compare the visual prognostic survival with and without MNV. The blue and red lines indicate the MNV- and MNV + groups, respectively. (**a**) Significant differences were observed between the MNV and MNV + groups in terms of the percentage of visual acuity loss of > 0 logMAR, with lower survival in the MNV + group. (*p* = 0.01). (**b**) The percentage of visual acuity loss of ≥ 0.1 logMAR was similar in the two groups (*p* = 0.059). (**c**) The prognosis of the MNV + group was poorer than that of the MNV- group in terms of the percentage of patients with visual acuity loss of ≥ 0.2 logMAR (*p* = 0.043). (**d**) No significant differences were observed between the MNV + and MNV- groups in terms of visual acuity change of ≥ 0.3 logMAR (*p* = 0.37). *MNV* macular neovascularization, *logMAR* logarithm of the minimum visual angle.

This study included 280 eyes of 236 consecutive patients, some of whom had bilateral involvement. Table 1 presents the patient characteristics. The mean follow-up period and cumulative duration of the persistence of SRD were 48.3 ± 43.3 months and 4.20 ± 11.64 months, respectively. Among the 280 eyes, 28 (10%) developed MNV during the observation period and were reclassified into the MNV(+) group. Of these, 27 eyes received treatment (17 received anti-VEGF, 10 PDT + anti-VEGF.) following the diagnosis of MNV. The remaining one eye showed spontaneous resolution of SRD after MNV development and was managed without treatment. The remaining 252 eyes did not develop MNV and remained completely untreated throughout the follow-up period, forming the MNV(−) group. The mean time to the onset of MNV was approximately 71 months (5.9 years), and the mean age at the time of onset was 70.6 ± 13.2 years. The patients in the MNV(+) group were older than those in the MNV(-) group at the time of the first visit (*p* = 0.0072, Mann–Whitney U test). Furthermore, the logMAR VA of the patients in the MNV(+) group was poorer, and the DLS rates at the time of the first visit were higher (Table 2). Figure 1 illustrates the Kaplan–Meier survival analysis comparing visual prognosis between the MNV(+) and MNV(-) groups. A significant difference was found in the percentage of visual acuity loss of > 0 logMAR (*p* = 0.01, Fig. 1a). No significant difference was found for the percentage of visual acuity loss of ≥ 0.1 logMAR (*p* = 0.059, Fig. 1b), whereas a significant difference was observed for ≥ 0.2 logMAR (*p* = 0.043, Fig. 1c). However, no significant difference was noted for ≥ 0.3 logMAR (*p* = 0.37, Fig. 1d).

**Table 1:** Background factors for all CSCR are presented.

Number (eyes)	280
Age (range)	55.3 ± 13.9 (23–87)
Male/Female	178/102
LogMAR VA at baseline	0.06 ± 0.26
Cumulative time of SRD existence (months)	4.20 ± 11.64
Baseline CCT (µm)	333 ± 109
The mean follow-up period (months)	48.3 ± 43.3
Eyes with DLS at baseline (eyes)	86
Eyes with DLS of length ≥ 1000 μm and height < 100 μm (eyes)	48

*CSCR* central serous chorioretinopathy, *logMAR* logarithm of minimal angle of resolution, *SRD* serous retinal detachment, *CCT* central choroidal thickness, *DLS* double layer sign.

**Table 2:** Characteristics of patients in the MNV(-) and the MNV(+) group.

	MNV(-)	MNV(+)	*P* value
Number (eyes)	252	28	
Age (range)	54.5 ± 13.9	62.0	0.0072*
Male/female (number of eyes)	162/90	16/12	0.48^†^
LogMAR VA at baseline	0.05 ± 0.23	0.18 ± 0.44	0.0071*
Baseline CCT (µm)	335 ± 109	317 ± 114	0.40^†^
Eyes with DLS (%)	72 (29)	14 (50)	0.02^†^
Eyes with DLS of length ≥ 1000 μm and height < 100 μm (eyes)	36 (14)	12 (43)	0.0001^†^

Values are presented as the mean ± standard deviation, unless otherwise indicated.

*CSCR* central serous chorioretinopathy, *MNV* macular neovascularization, *logMAR* logarithm of minimal angle of resolution, *SRD* serous retinal detachment, *CCT* central choroidal thickness, *DLS* double layer sign, *SIRE* shallow irregular RPE elevation.

*Linear Mixed Models. ^†^Chi-squared test.

Cox proportional hazards analysis performed to identify the factors associated with the development of MNV confirmed that the persistence of SRD for ≥ 6 months (odds ratio 4.3, 95% confidence interval 1.75–12.23; *p* = 0.0027) and the presence of DLS measuring ≥ 1000 μm in length and < 100 μm in height (odds ratio 2.44, 95% confidence interval 1.02–5.69; *p* = 0.0405) exhibited significant associations with the development of MNV (Table 3).

**Table 3:** Factors associated with MNV development following CSCR.

	Univariate analysis	Multivariate analysis		
Variable	P value	Coefficient	SE	P value
Age	0.027	NS		
Sex	0.24	NS		
CCT	0.41	NS		
Baseline logMAR	0.025	NS		
The presence of DLS	0.055	NS		
The presence of DLS of length ≥ 1000 μm and height < 100 μm	0.0042	− 0.73	0.24	0.041
The cumulative time of SRD existence for ≥ 6 months	< 0.0001	− 0.45	0.22	0.0027

*CSCR* central serous chorioretinopathy, *MNV* macular neovascularization, *CCT* central choroidal thickness, *DLS* double layer sign, *SRD* serous retinal detachment, *NS* not selected.

**Fig. 2:**
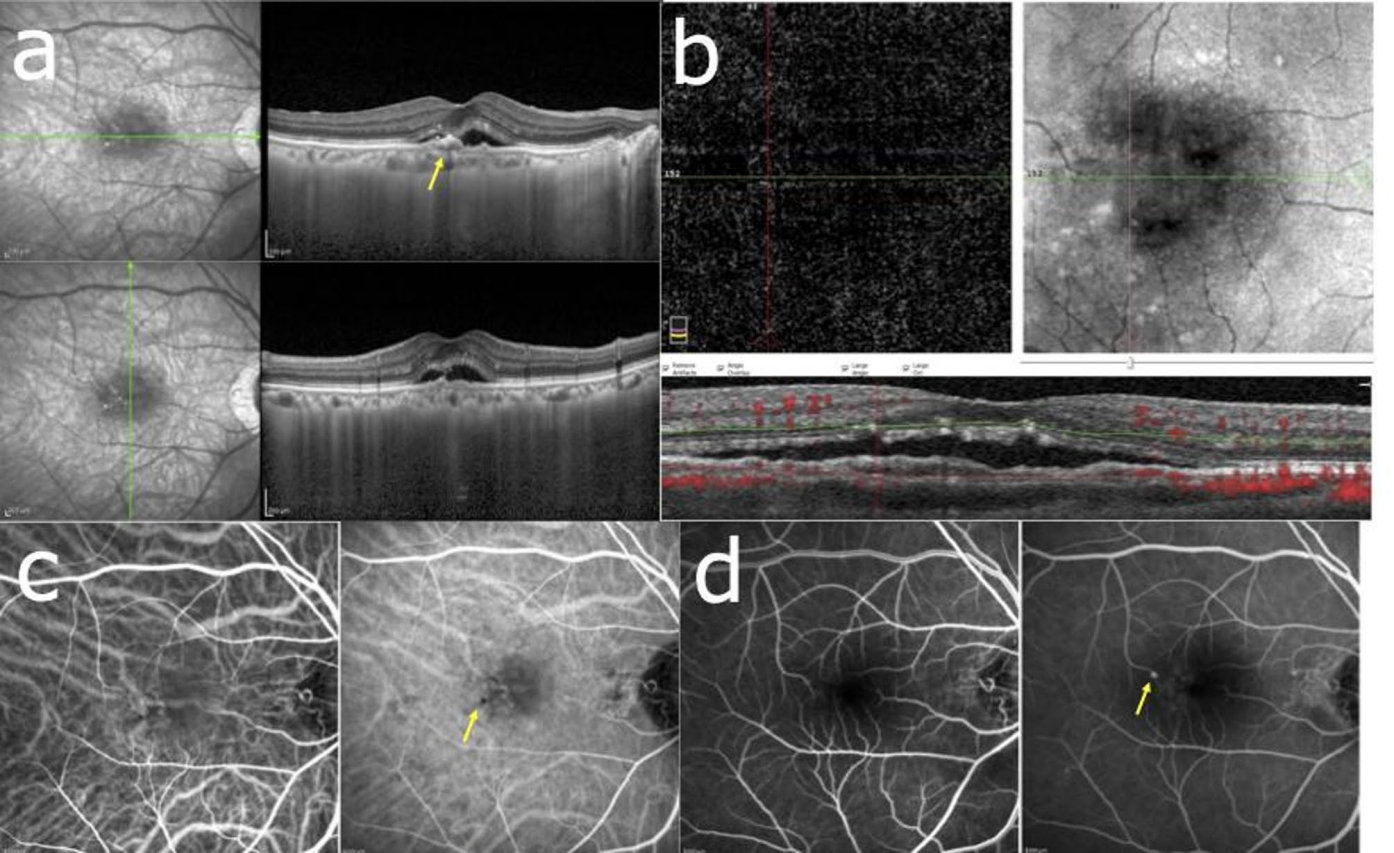
Case presentation. A 75-year-old woman with visual acuity Vd=(1.2) at initial examination. (**a**) OCT shows the presence of a DLS of ≥ 1000 μm in length and < 100 μm in height and SRD. (**b**) OCTA shows no obvious flow signal in the outer retina. (**c**) No obvious macular neovascularization in (left) early and (right) late ICGA. (Right) Slight CVH findings (yellow arrow) on late ICGA. (**d**) (left to right) Leak point (yellow arrow) observed from early to late FA. *OCT* optical coherence tomography, *OCTA* optical coherence tomography angiography, *FA* fundus angiography, *ICGA* indocyanine green fluorescence fundus angiography.

**Fig. 3:**
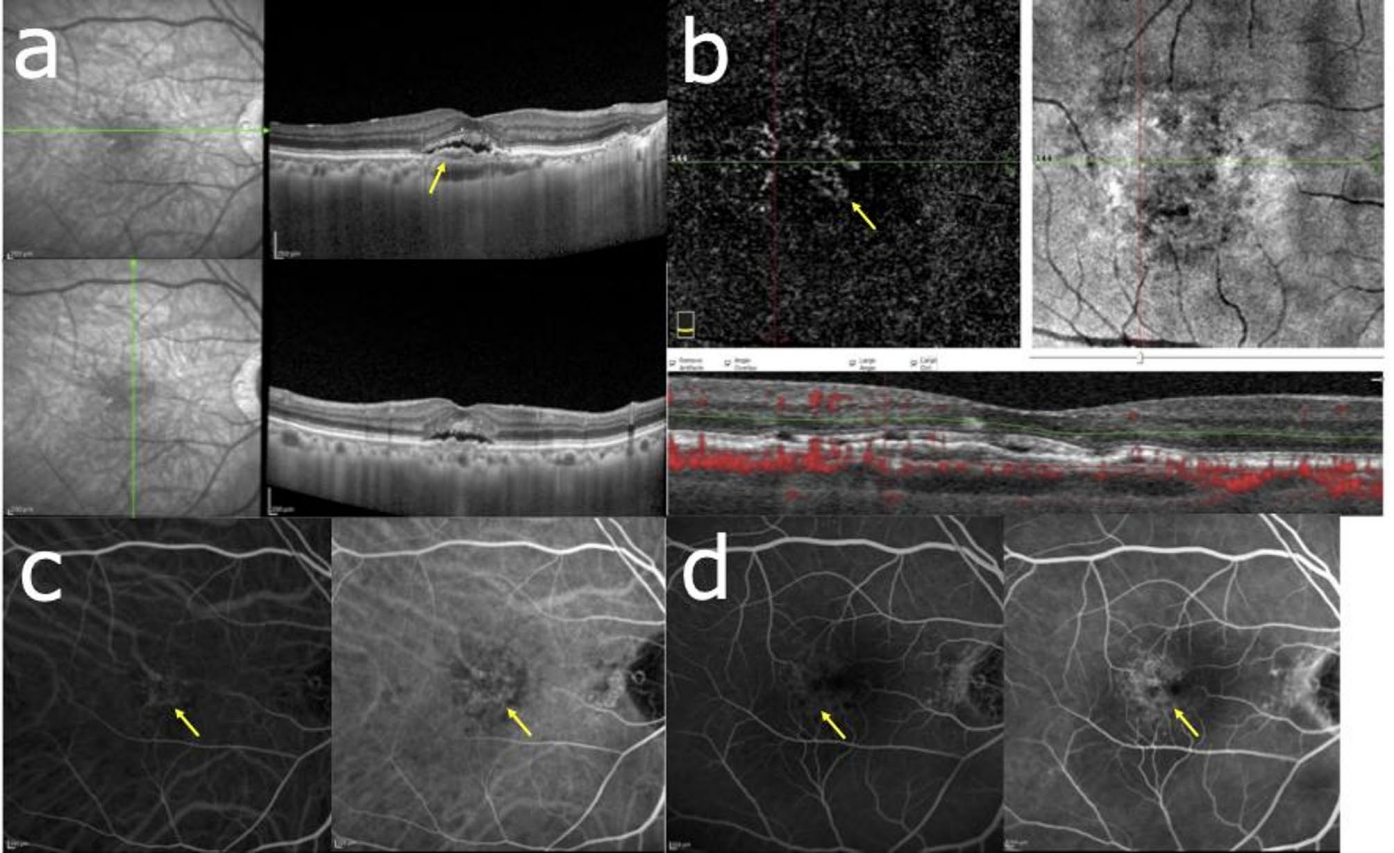
Two-year follow-up. Visual acuity Vd=(1.0). (**a**) OCT shows the presence of a DLS (yellow arrow) that is ≥ 1000 μm in length and < 100 μm in height and SRD. (**b**) OCTA shows flow signal (yellow arrow) in the outer retina. (**c**) Left) Suspected macular neovascularization in early ICGA; Right). Slight macular neovascularization findings (yellow arrows) in late ICGA. (**d**) (left to right) Occult CNV findings from early to late FA. *OCT* optical coherence tomography, *OCTA* optical coherence tomography angiography, *FA* fundus angiography, *ICGA* indocyanine green fluorescence fundus angiography.

The inter-observer agreement for the identification of DLS was excellent, with a Cohen’s κ value of 0.95. Figure 2 presents a representative case of a 75-year-old patient with DLS (≥ 1000 μm length, < 100 μm height) and SRD at baseline (Fig. 3a). OCTA and ICGA & FA findings confirmed the absence of MNV at initial presentation (Fig. 2b–d). Figure 3 illustrates the two-year follow-up of the case shown in Fig. 2. MNV development was observed, with OCTA showing a flow signal in the outer retina (Fig. 3b) and ICGA & FA revealing neovascularization features (Fig. 3c, d).

**Fig. 4:**
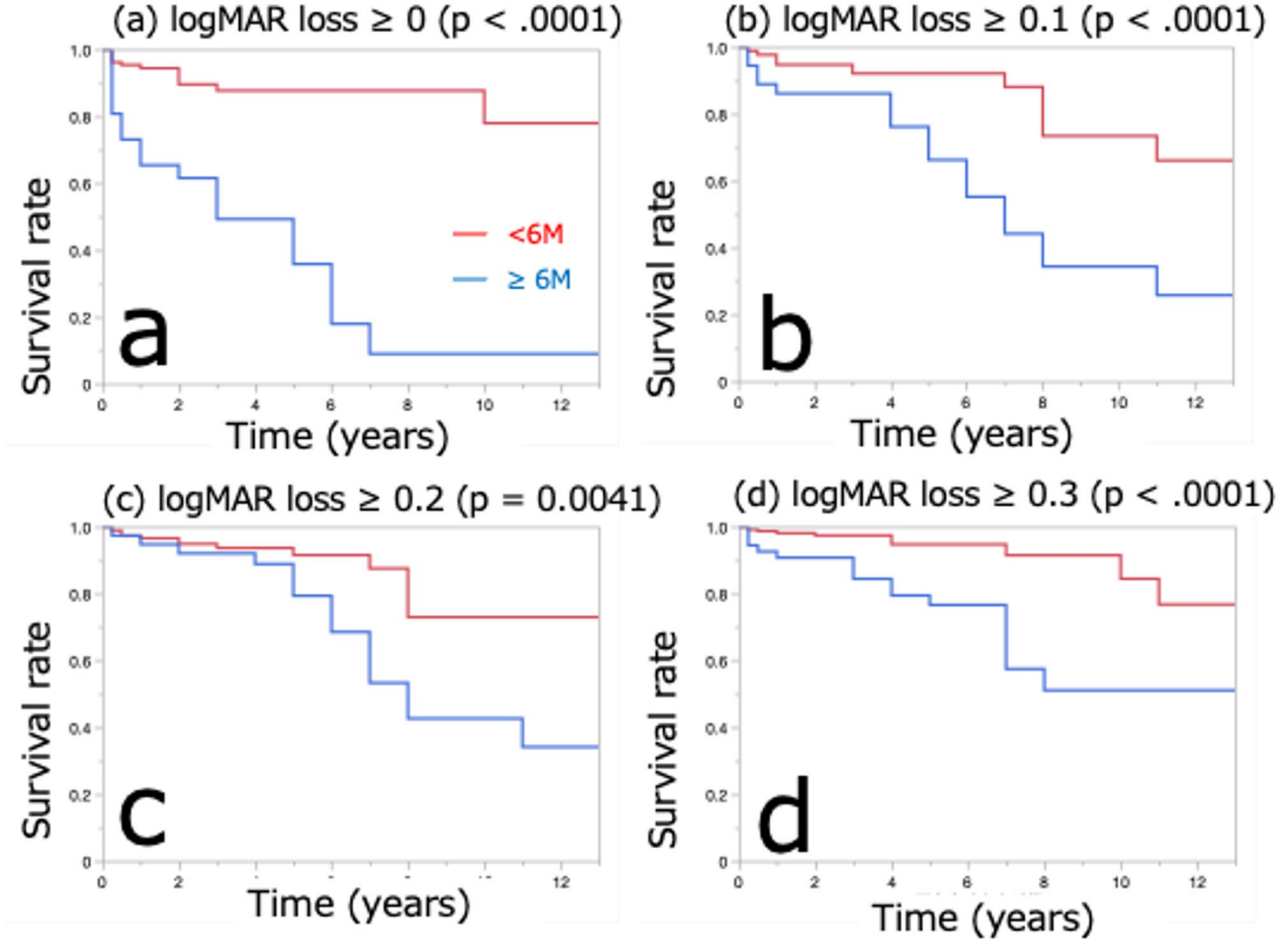
The Kaplan–Meier method was used to compare visual prognostic survival according to they cumulative duration of the presence of subretinal fluid (< 6 months or > 6 months). The red line indicates the < 6 months group, whereas the blue line indicates the > 6 months group. (**a**) Percentage of patients with visual acuity loss of ≥ 0 logMAR (*p* < 0.0001). (**b**) Percentage of patients with visual acuity loss of ≥ 0.1 logMAR (*p* < 0.0001). (**c**) Percentage of patients whose visual acuity decreases by ≥ 0.2 logMAR (*p* = 0.0041). (**d**) Visual acuity change of ≥ 0.3 logMAR (*p* < 0.0001). For both visual acuity criteria, a significant difference was observed between the cumulative duration of the presence of subretinal fluid at < 6 and > 6 months. *logMAR* logarithm of the minimum visual angle.

Cox proportional hazards regression analysis performed to evaluate the factors associated with changes in VA during the observation period revealed that the cumulative duration of the persistence of SRD was the only factor exhibiting significant associations with visual outcome in patients with CSCR (odds ratio 4.7, 95% confidence interval: 2.02–11.37, *p* = 0.0009, Table 4). Figure 4 shows the Kaplan–Meier analysis comparing visual prognosis based on SRD duration (< 6 months vs. >6 months). Patients with SRD persisting for more than 6 months exhibited significantly worse visual prognosis across multiple logMAR criteria (Fig. 4a–d).

**Table 4:** Factors associated with visual decline greater than 0.3 logmar.

	Univariate analysis	Multivariate analysis		
Variable	P value	Coefficient	SE	P value
Age	0.042	NS		
Sex	0.19	NS		
CCT	0.24	NS		
Later MNV development	0.41	NS		
The presence of DLS	0.66	NS		
The presence of DLS of length ≥ 1000 μm and height < 100 μm	0.60	NS		
The cumulative time of SRD exeistence for ≥ 6 months	0.0002	− 0.74	0.20	0.0006

*CSCR* central serous chorioretinopathy, *MNV* macular neovascularization, *CCT* central choroidal thickness, *DLS* double layer sign, *SRD* serous retinal detachment, *NS* not selected.

## Discussion

The present study revealed that 28 (10%) of the 280 patients with untreated CSCR developed MNV during a mean follow-up period of 48.3 ± 43.3 months. Multivariate analysis revealed that the cumulative duration of the persistence of SRD and the presence of DLS measuring ≥ 1000 μm in length and < 100 μm in height were associated with the development of MNV.

Chronic CSCR has been associated with the development of MNV^9^. Previous studies exploring the prevalence of MNV in patients with CSCR reported the development of MNV during the follow-up period in 2–9% of patients^19^. Consistent with these findings, 10% of cases developed MNV during the follow-up period in the present study. The mean time to the development of MNV in patients with untreated CSCR was approximately 5.9 years in the present study. Few studies have examined the time from the initial diagnosis of CSC to the development of MNV. Kim et al. reported a mean duration of 1.65 years between the initial diagnosis of CSC and the development of MNV.^20^ In contrast, Mrejen et al. reported that the time from the diagnosis of CSC to the detection of CNV was 17.0 years^21^. However, most patients included in these reports had received PDT and other therapies for CSCs, and the time to the development of MNV in patients with untreated CSCR was unknown. Furthermore, the study conducted by Mrejen et al.^21^ focused on the period from 1977 to 2018, and the widespread use of OCTA may have contributed to the early detection of MNV in the study by Kim et al.^20^ The mean time to the detection of MNV was 5.9 years in patients with untreated CSC in the present study. However, this was a single-center, retrospective study, and the characteristics of the patients may have differed from those included in their study. Further prospective studies must be conducted to identify the factors that may have influenced the differences in the mean time to the development of MNV.

The presence of DLS measuring ≥ 1000 μm in length and < 100 μm in height exhibited a significant association with the development of MNV in the present study. DLS indicates the focal elevation of the RPE and may reflect early pathological changes, including nonexudative MNV^16,22,23^. The presence of MNV in lesions of ≥ 1000 μm in length and < 100 μm in height can be detected with high sensitivity and specificity on OCTA^18^. However, all DLS do not contain MNV. The findings of the present study characterized DLS that did not contain MNV but were at high risk of developing MNV in patients with CSCR. We acknowledge the importance of standardization and reproducibility in defining DLS. The dimensional thresholds used in this study (≥ 1000 μm in length and < 100 μm in height) were not arbitrarily chosen but were based on previously published criteria for shallow irregular RPE elevation (SIRE), as proposed by Narita et al.^23^ and referenced in the work of Csincsik et al.^24^. Furthermore, we confirmed the reliability of our assessments through inter-observer agreement analysis, which demonstrated excellent reproducibility (Cohen’s κ = 0.95). Notably, non-numerically defined DLS did not exhibit a significant association with the development of MNV. This may be attributed to DLS including other non-neovascular causes of RPE elevation, such as drusen, dPED, and sPED. A more detailed characterization of DLS for the detection or prediction of the development of MNV in the future will aid in the management of CSCR or entire pachychoroid spectrum diseases.

The present study revealed that the cumulative duration of the persistence of SRD exhibited a significant association with the development of MNV, suggesting that this was an important predictor of MNV in patients with untreated CSCR. This finding indicates that the persistence of SRD, even if intermittent, may result in RPE dysfunction and induce the development of choroidal angiogenesis^8,18^. Persistent SRD disrupts the barrier function of the RPE, thereby promoting the disruption of the blood-retinal barrier^4^. Furthermore, the persistence of SRD increases the mechanical load on the BM and RPE. This induces the local overproduction of VEGF, which may contribute to the development of MNV^25,26^. The present study revealed the impact of SRD on the development of MNV by specifically quantifying the cumulative duration of the persistence of SRD. Thus, determining the cumulative duration of the persistence of SRD, even after its resolution, is important in the management of CSCR. Patients with cumulative duration of SRD persistence of ≥ 6 months must be assessed carefully. In addition, assessment of the relationship between the duration of SRD and VEGF levels or function of the RPE might contribute to understanding its pathogenesis.

Recent advances in multimodal imaging have also underscored the importance of choroidal sublayer analysis in pachychoroid spectrum diseases. In particular, Viggiano et al. recently demonstrated that the relative proportions of Haller’s and Sattler’s layers beneath flat irregular pigment epithelial detachments (FIPEDs) differ significantly between CSCR eyes with and without MNV, suggesting a role of choroidal architecture in disease progression^27^. Although our present study did not include choroidal layer-specific analysis due to the retrospective nature of the dataset spanning from 2009 to 2022 and variability in imaging protocols, this line of investigation represents a promising future direction. Prospective studies incorporating standardized imaging protocols and automated sublayer segmentation may offer deeper insights and improve risk stratification in CSCR-related MNV.

Moreover, our findings should be interpreted within the conceptual framework of the pachychoroid disease spectrum. As recently reviewed by Viggiano et al.^28^, CSCR, pachychoroid neovasculopathy (PNV), and polypoidal choroidal vasculopathy (PCV) share common choroidal alterations and likely represent stages within a spectrum of disease progression. The cumulative duration of SRD and the presence of DLS, as demonstrated in our study, may reflect this progressive choroidal dysfunction. Future studies integrating multimodal imaging—including OCTA and en face analysis—will further elucidate how pachychoroid phenotypes predispose to MNV.

Patients with CSC exhibited poor visual prognosis during long-term follow-up^21,29^. In particular, significantly poorer VA was observed in patients with CSC accompanied by MNV^9^. In the present study, the changes in VA during the period before the development of MNV were similar to those observed in the patients who did not develop MNV in the present study. Thus, the pre-stage of MNV development may have no impact on VA. In contrast, a cumulative duration of the persistence of SRD of > 6 months was significantly associated with poorer visual prognosis during observation. Thus, treatment interventions should be considered at an appropriate time in patients with persistent SRD to improve visual prognosis. The incidence of MNV has been reported to increase after laser therapy or PDT for CSCs; consequently, careful observation should be continued to monitor vision loss owing to the development of MNV, even if SRD resolves with treatment^12,13^. The optimal timing of treatment of persistent SRD should be determined after considering the increased risk of vision loss and development of MNV due to persistent SRD and the increased risk of development of MNV owing to treatment. Determining the course of CSCR after treatment was beyond the scope of this study. Prospective studies must be conducted in the future to address these issues.

The present study provides new insights by using the cumulative duration of the persistence of SRD in the analysis; however, certain limitations exist. First, choroidal sublayer analysis (e.g., Haller’s and Sattler’s layers) was not performed due to the retrospective design of the study and the lack of standardized enhanced-depth imaging across the extended study period. Second, the relatively small number of eyes that developed MNV (*n* = 28) may limit the statistical power of the multivariate analysis. Furthermore, as this was a single-center study conducted in a Japanese cohort, the findings may not be generalizable to broader populations, given known ethnic and regional differences in pachychoroid spectrum diseases. Prospective studies with larger sample sizes must be conducted as this was a retrospective study design with a non-standardized follow-up protocol. Despite these limitations, this study has several strengths, including a relatively large sample size, detailed multimodal imaging findings, and long-term follow-up. The detailed analysis of the cumulative duration of the persistence of SRD underscores the importance of close monitoring and accurate data collection for the management of CSCR. Although choroidal sublayer analysis was not feasible, we believe that our findings on SRD duration and DLS dimensions offer valuable insights within the context of the available data.

In conclusion, the present study revealed that the cumulative duration of the persistence of SRD and numerically defined DLS are important factors associated with the development of MNV in patients with untreated CSCR. These findings emphasize the importance of early detection and commencement of therapeutic intervention for MNV, especially in patients with persistent and intermittent subretinal fluid accumulation. Individualized management strategies must be developed to reduce the risk of visual impairment during long-term follow-up.

## Methods

### Study design

This retrospective study adhered to the principles of the Declaration of Helsinki and was approved by the Institutional Review Board of the University of Tokyo (approval no. 2217). The requirement for obtaining written informed consent was waived by the Institutional Review Board owing to the retrospective design of the study. Patients who did not consent to the review of their medical records for research purposes were excluded.

### Participants

The medical records of consecutive patients with untreated CSCR who visited the University of Tokyo Hospital between June 2009 and April 2022 and were followed up for at least 6 months were examined retrospectively. Standard ophthalmologic examinations, including the measurement of corrected visual acuity (VA) using the Landolt C optotype, slit-lamp microscopy, fundus examination, and spectral-domain optical coherence tomography (SD-OCT; Spectralis, Heidelberg Engineering), were conducted at each visit. The presence of MNV was ruled out during the initial visit using OCTA after 2016. The corrected VA was measured as decimal VA and converted to the logarithm of the minimum visual angle (logMAR). All patients, except those with contraindications, underwent fluorescence fundus angiography (FA) and indocyanine green fluorescence fundus angiography (ICGA) during the initial visit.

Patients without SRD at the time of the initial examination and those with other retinal diseases, such as diabetic retinopathy, retinal vascular disease, myopic maculopathy, glaucoma, or severe cataracts, that impair visual function were excluded from the analysis.

The OCTA and/or FA and ICGA findings were used to define the development of MNV. Patients with MNV detected at the time of the initial visit were excluded.

### Imaging evaluation

The presence of DLS was assessed using OCT B-scan images obtained at the baseline visit. Only baseline OCT findings were used for DLS classification to maintain consistency and avoid temporal bias during the longitudinal follow-up.

DLS was defined as non-uniform reflectance within the irregular ridges of RPE based on the existing criteria for OCT findings^17^. DLS findings that fulfilled specific dimensional criteria—horizontal length ≥ 1000 μm and maximal height < 100 μm—on B-scan OCT images were extracted as described in previous reports^23^. These dimensional thresholds were based on prior literature describing shallow irregular RPE elevations (SIRE), originally defined by Narita et al. and cited by Csincsik et al.^23,24^. To assess reproducibility, two independent graders (masked to clinical outcomes) evaluated the presence of DLS. Inter-observer agreement was quantified using Cohen’s kappa coefficient (κ). The cumulative duration of SRD was determined by reviewing the serial OCT images and clinical records throughout the follow-up period. Periods of continuous SRD presence were summed to calculate the total duration, as described in previous studies^8^.

### Follow-up

Patients diagnosed with CSC without MNV at the time of the initial examination were followed up. Patients who exhibited spontaneous resolution of the SRD were followed up regularly without treatment. Patients with SRD persisting for > 2 months received treatment after providing consent. Follow-up was discontinued once the patients were treated. OCTA, FA, or ICGA was performed to confirm the development of MNV if a clear deterioration of fundus examination and OCT findings was observed; observation was terminated if MNV was detected.

### Statistical analysis

Demographic factors, such as age at the time of the first CSCR visit, sex, and age at the time of MNV detection, were included in the analysis. Patients who did and did not develop MNV during the follow-up period were included in the MNV(+) and MNV(-) groups, respectively.

Cox proportional hazards analysis was performed using age, sex, central choroidal thickness (CCT), presence of DLS or DLS of ≥ 1000 μm in length and < 100 μm in height, and cumulative duration of SRD persistence of > 6 months as explanatory variables to identify the factors predicting the development of MNV. The development of MNV was defined as the event. Multivariate analysis was conducted using only explanatory variables with p-values of < 0.05 after analyzing each explanatory variable.

A logMAR VA loss of ≥ 0.3 was defined as the event. Multivariate analysis was conducted using only explanatory variables with p-values of < 0.05, after analyzing each explanatory variable.

All statistical analyses were performed using JMP software (version 16.0, SAS Institute, Cary, NC, USA). A p-value of < 0.05 was considered statistically significant. Data are presented as mean ± standard deviation.

## Data Availability

The datasets generated and/or analyzed in this study will be made available by the corresponding author upon reasonable request.
